# Facile and green synthesis of α-Fe_2_O_3_ nanoparticles stabilized with chitosan for phototherapy with 808 nm laser irradiation

**DOI:** 10.1038/s41598-025-17797-2

**Published:** 2025-09-02

**Authors:** Farshad Dehdashti, Hossein Shirkani, Mohsen Mehrabi, Amirhossein Ahmadi

**Affiliations:** 1 Physics Department, Persian Gulf University, P. O. Box: 7516913817, Bushehr, Iran; 2 Department of Biological Science and Technology, Persian Gulf University, P. O. Box: 7516913817, Bushehr, Iran

**Keywords:** Green synthesis, α-Fe_2_O_3_ of nanoparticles, Chitosan, Nanocomposite, Photothermal therapy, Photodynamic therapy, Biotechnology, Chemistry, Materials science, Nanoscience and technology, Optics and photonics

## Abstract

In this research, biocompatible α-Fe_2_O_3_ nanoparticles were prepared as an agent for photothermal and photodynamic therapy methods by combining green synthesis and hydrothermal methods. The addition of chitosan bio-polymer played a crucial role in this process, as it not only stabilized the suspension of nanoparticles but also enhanced their biocompatibility. This stability was confirmed by zeta potential analysis Various analyses such as transmission electron microscope, X-ray diffraction, UV–visible spectrum, and Fourier transform infrared spectrum were performed to determine the structural and optical characteristics of the nanocomposite. The average size of the spherical crystals of α-Fe_2_O_3_ nanoparticles was estimated at 43 nm using the Williamson–Hall equation. The corresponding band gap value of nanoparticles was estimated at 1.8 eV by drawing a Tauc diagram. Photothermal effects for several different concentrations of an aqueous solution of CS-nanocomposite were measured by an 808 nm laser with a power density of 1 W/cm^2,^ and the concentration of 5 mg/ml was chosen as the optimal concentration for use in photothermal therapy. The value of the photothermal conversion efficiency of this nanocomposite was determined at 7% using Roper’s equation. To investigate the photodynamic properties of nanoparticles, a methylene blue probe was used to detect active oxygen species. Finally, an MTT assay studied the cytotoxicity of nanocomposite on AGS cells before and after laser irradiation. Under laser irradiation, cell viability at concentrations of 250ppm and 500ppm was 88% and 69%, respectively, compared to the control cells, confirming this nanocomposite’s photothermal therapy and photodynamic therapy effects.

**Supplementary Information:**

The online version contains supplementary material available at 10.1038/s41598-025-17797-2.

## Introduction

These days, cancer is one of the most common diseases that affects many people’s lives^1^. In this disease, some normal cells grow uncontrollably; if the diagnosis is delayed, these abnormal cells can progress throughout the body^2^. There are many treatment methods, including chemotherapy, radiation therapy, surgery, etc., for cancer treatment, and sometimes, based on the patient’s condition, one method or several methods simultaneously are used. In these conventional techniques, some healthy cells are also unintentionally destroyed, in addition to cancer cells, which is one of the most significant disadvantages of the treatment methods mentioned above. Therefore, a non-invasive treatment method, which refers to a procedure that does not involve the insertion of an instrument or device into the body, is vital^3–5^. In 1959, for the first time, Richard Feynman presented a conceptual model of nanotechnology^6^. At dimensions larger than approximately 100 nm, the behavior of materials can be accurately described by classical physics, whereas at scales below 100 nm, quantum mechanical effects become significant and must be considered to properly describe material properties. Materials in nanometer dimensions can increase ion transport and, as a result, increase reactivity in their surrounding environment due to their high surface-to-volume ratio^7^. Fortunately, with the emergence of nanotechnology and significant investments in this field, various photo-nano-medicines with unique physical, chemical, and biological properties have been produced in recent years. New non-invasive treatment methods, including photothermal therapy (PTT) and photodynamic therapy (PDT), have been presented, which have reduced many of the disadvantages of conventional treatment methods. In PTT, nanoparticles (NPs) adjacent to cancer cells are exposed to laser or other light sources with a specific wavelength. In the meantime, the energy of electromagnetic radiation is converted into heat energy by using NPs; as a result, these cells die with the increase of temperature up to 43°C^8^. In PDT, NPs accumulated on cancerous tissues are exposed to light with a specific wavelength. After interaction with light and in the presence of oxygen molecules in a certain amount around these NPs, they produce reactive oxygen species (ROS) and then cause the death of cancer cells^9^. In these light-based treatment techniques, electromagnetic waves in the infrared range usually are used because in this range, the absorption and scattering of light by the human body has its minimum value^10^. Unlike conventional treatment methods, cancerous tissues are precisely targeted in these methods. Furthermore, not only were the side effects caused by the treatment method greatly reduced, but the effectiveness of the treatment method was also significantly increased. Therefore, by choosing suitable biocompatible NPs, these two treatment techniques can be used simultaneously, and the side effects, the duration of the treatment period, and the treatment costs can be significantly reduced. Up to now, many biocompatible NPs have been used as agents for PTT and PDT. In previous studies, the ability of calcium fluoride NPs, graphene-silica-quantum dots nanocomposite, and copper sulfide NPs to create ROS was shown by using an anthracene probe^11–13^. Although various metal NPs, including gold (Au), palladium (Pd), silver (Ag), and platinum (Pt) NPs, have a high ability to absorb electromagnetic radiation, because of their high cost, iron oxide, copper oxide, nickel oxide, and titanium oxide NPs have been replaced^14^. Meanwhile, Fe_2_O_3_ NPs have attracted the attention of many researchers due to their unique optical, magnetic, chemical, and bioavailability properties. The distinct properties of Fe_2_O_3_ NPs, such as their optical, magnetic, and chemical characteristics, make them a promising candidate for further research. The shape and size of Fe_2_O_3_ NPs play an important role in cytotoxicity, and these parameters can be controlled during synthesis. Besides, various parameters such as nanoparticle size, surface charge, core composition, and coating properties of NPs effectively affect their dispersion^15^. Other advantages of using Fe_2_O_3_ NPs include the high ability to absorb electromagnetic waves in the infrared region to use PTT, the ability to produce ROS to apply PDT, their non-toxicity and exhibiting paramagnetic properties when their size ranges between 20 and 30 nm, enabling them to be guided toward cancerous tissue by applying a suitable external magnetic field^8– 16^. Fe_2_O_3_ NPs can be produced using different methods such as hydrothermal, co-precipitation, etc^17–19^. Researchers worldwide are looking for a low-cost synthesis method without high temperature and pressure, compatible with the environment, and capable of mass production. In recent years, the green synthesis method for producing photo-nano-medicines has received much attention. This new synthesis method has been an excellent alternative to conventional chemical methods. In this technique, extracts of almost natural plants are used as a reducing agent and improving the biocompatibility of NPs^20^. One of the significant problems of NPs is their tendency to agglomerate in colloidal suspensions, which leads to instability. The interaction between NPs dissolved in a liquid medium occurs successively, and the stability of such suspensions is based on these interactions. Electrostatic forces between NPs play an essential role in the stability of a suspension. The van der Waals force between NPs is one of the main reasons for agglomeration^21^. In order to neutralize such an attractive force between NPs and create a stable suspension, the need for a short-range repulsive force can be a solution. Iron oxide NPs can be dissolved in an aqueous solution through specific interactions between the surfaces of NPs and polymers with low molecular weight and create a stable suspension^22^. Meanwhile, Chitosan (CS), a natural substance, can stabilize suspensions containing NPs effectively. Chitin can be extracted from aquatic animals such as crabs, mushrooms, and insects^23–25^. By separating chitin acetate groups, biopolymer CS is obtained, which is very interesting in cancer treatment due to its high biocompatibility and biodegradability, impressive biological activities, and low-cost production^26^. In recent years, many studies have been conducted on the medical applications of iron oxide NPs. In 2014, Alarifi showed the destruction of mitochondrial organelles and the subsequent death of breast cancer cells with the help of combining anti-cancer drugs with iron oxide NPs^27^. In 2017, Essa and his colleagues synthesized iron oxide NPs using polyphenolic compounds found in grape leaves (Vitis vinifera). They reported high cytotoxic effects against B20L cells at 10 and 5 mg/L concentrations, inhibiting cancer cell growth by 70.5 and 5.8%, respectively^28^. In 2020, Sandhya synthesized iron oxide NPs by green synthesis using Palmyra palm seed membrane extract. These NPs showed high biocompatibility with NIH 3T3 cells at 50–500 µg/ml concentrations. This type of synthesis increased therapeutic properties and the biocompatibility of NPs. Also, these NPs showed significant antimicrobial and antioxidant activity^29^. In another study in 2021, Fe_2_O_3_ NPs with folic acid loaded with doxorubicin were used to treat a type of lymphoma cancer cells. In the vivo assay, the results showed that the reduction in tumor size in mice that received NPs containing 5 µg of doxorubicin was more significant than in mice that did not receive doxorubicin^30^. In recent years, researchers in the field of cancer treatment have produced Fe_2_O_3_ NPs using the green synthesis method using the extract of Chlorella Vulgaris plant, orange peel, pepper leaf and lemon peel, etc^31–34^. In this study, green tea extract was employed as a natural source of polyphenols for the synthesis of α-Fe_2_O_3_ NPs used in PTT and PDT. A combination of green synthesis and hydrothermal processing was utilized to minimize the use of hazardous chemicals and maintain environmental sustainability. The polyphenols present in the extract, including epigallocatechin-3-gallate (EGCG) and gallocatechin gallate (GCG), acted as surfactants and became deprotonated under elevated temperature and pressure. These molecules subsequently interacted with hydrolyzed iron(III) species, leading to the formation of intermediate complexes. The partially hydrophobic nature of these complexes facilitated aggregation. Upon further temperature increase during the hydrothermal process, the iron species underwent a phase transformation into primary α-Fe_2_O_3_ NPs, which rapidly aggregated due to their high surface energy, resulting in the formation of a porous structure^3^. To improve stability, we utilized CS as a bio-polymer stabilizer. The resulting NPs were systematically evaluated for their structural, optical, photothermal, and photodynamic properties, as well as their light-induced toxicity in vitro.

## Materials and methods

### Materials

Iron nitrate (Fe(NO_3__3_·9H_2_O) (≥ 99.0% ), methylene blue (C_16_H_18_CIN_3_*S*) (dye-content ≥ 95.0% ) and acetic acid CH_3_COOH (≥ 99.0–100% ) from Merck company, CS (C_6_H_11_NO_4_) (degree of deacetylation: 75–85%) from Sigma-Aldrich company and green tea leaves from Iran were prepared. The AGS human gastric adenocarcinoma cell line was purchased from the Iranian Biological Resource Center (IBRC. Tehran, Iran) and also MTT assay kit (BioIdea, Tehran, Iran) was used for evaluating cell viability.

### Equipment

Analysis transmission electron microscope (TEM) (Philips EM 208 S) and X-ray diffraction (XRD) (BRUKER D8 Advance Kα-Cu) were used to determine the shape and size of crystals and the degree of crystallinity. NPs and nanocomposites infrared spectra were measured using an FTIR spectrometer, model FT/IR 4600 (JASCO, a company made in Japan), to identify the functional groups on their surface. The UV–Vis absorption spectra were recorded in a UV–visible spectrophotometer model 250 SPECORD, made in Germany. Zeta potential analysis was performed by CAD Instrument Company (Zeta Compact model) to check the stability of the suspension containing NPs and nanocomposites. A specialized spectrophotometer from Biotek company (Synergy HTX (gen5) model) made in America was used to measure the absorbance of the reaction product created in the MTT assay. An 808 nm near-infrared diode laser with a power density of 1 W/cm^2^ as a radiation source and a temperature-measuring sensor (XH-W3001) was also used to measure the temperature.

### Preparation of CS-α-Fe_2_O_3_ nanocomposite

#### Green tea extract preparation

First, the green tea leaves were washed with deionized (DI) water and placed in an oven at 70 °C for 4 h. Then, 250 ml of DI water was added to 3.33 g of dried green tea leaf powder and placed on a magnetic stirrer at 80 °C for 20 min. The tea extract solution was filtered, and finally, it was obtained^35^.

#### Synthesis of α-Fe_2_O_3_ NPs

In a beaker, 15 ml of green tea extract was dissolved in 50 ml of DI water, followed by 2.5 g of Fe(NO_3_)_3_·9H_2_O added to them and placed on a magnetic stirrer for 30 min at room temperature. The obtained solution was transferred to a 500 ml autoclave and was placed in a furnace at 180 °C for 13 h. The solution was centrifuged at 8000 rpm, and the product was washed several times. In the next step, the prepared powder was placed in an oven at 75 °C for 4 h, and finally, α-Fe_2_O_3_ NPs were obtained^35^.

#### Fabrication of CS-α-Fe_2_O_3_ nanocomposite

**Fig. 1:**
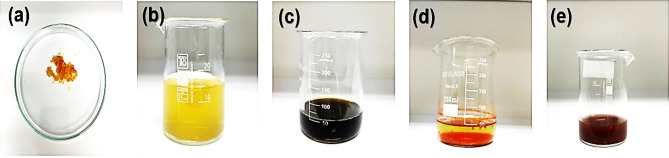
Synthesis process **a** iron nitrate, **b** tea extract, **c** iron nitrate solution dissolved in tea extract, **d** precipitation of α-Fe_2_O_3_ NPs, **e** CS-α-Fe_2_O_3_ nanocomposite.

100 mg of CS powder was dissolved in 5 ml of CH_3_COOH and placed on a magnetic stirrer for 5 h. Following this, 250 mg of α-Fe_2_O_3_ NPs powder was added to the above solution and placed on a magnetic stirrer at a speed of 700 rpm for 7 h. The solution was then placed in an oven and heated at 75 °C for 5 h. This systematic approach led to the formation of CS-α-Fe_2_O_3_ nanocomposite. Figure 1 provides a detailed schematic of the raw materials and the color change of the NPs solution during the synthesis process^36^.

### Photothermal effect

Different concentrations of CS-α-Fe_2_O_3_ nanocomposite solution (0.1, 0.5, and 2 mg/ml) were prepared using an ultrasonic device to measure PT effects. 3 ml of these different concentrations was poured into a glass tube and exposed to an 808 nm laser with a power density of 1 W/cm^2^. The increased temperature was measured in 15 min using a sensor (XH-W3001). Besides, by applying Roper’s equation and performing relevant experiments, the PT conversion efficiency of this nanocomposite was calculated.

### PD effect

This research used methylene blue as a probe to detect ROS around α-Fe_2_O_3_ NPs under an 808 nm laser with a power density of 1 W/cm^2^. In two separate breakers, similar methylene blue solutions, α-Fe_2_O_3_ NPs, and distilled water with specific concentrations were prepared, and one solution was exposed to a laser for 10 min. In the container under laser irradiation, the reaction of methylene blue with active oxygen species decreased its concentration in the solution, and this was determined by the decrease in the absorption spectrum of methylene blue in the breaker under 808 nm laser compared to the spectrum of methylene blue in the breaker without laser irradiation.

### In vitro cytotoxicity

To evaluate the cytotoxic effects of the CS-nanocomposite on AGS cells, an MTT assay was conducted using the BioIdea MTT assay kit (BioIdea, Iran), following the manufacturer’s instructions. This colorimetric method relies on the enzymatic reduction of yellow tetrazolium salt (MTT) to purple formazan crystals by mitochondrial succinate dehydrogenase in viable cells. AGS cancer cells (8,000 cells per well), obtained from the Iranian Biological Resource Center (IBRC), were cultured in 96-well plates and incubated overnight. The cells were then treated with the nanocomposite at concentrations of 250 and 500 ppm, while untreated cells served as controls. In addition, a subset of treated wells was exposed to an 808 nm laser to assess the combinatorial photothermal effect, while others were kept without irradiation. After 24 h of incubation, the culture medium was removed, and cells were washed with phosphate-buffered saline (PBS). Subsequently, 100 µL of RPMI-1640 medium and 10 µL of MTT solution (5 mg/mL) were added to each well, followed by incubation at 37 °C. After formazan crystal formation, 100 µL of lysis buffer was added to dissolve the crystals, and absorbance was measured at 570 nm using a BioTek ELISA reader. The assay was performed in three independent biological replicates, each with five technical replicates. Relative cell viability was calculated by comparing absorbance values of treated samples to untreated controls. Statistical analysis was conducted using one-way ANOVA with Tukey’s post hoc test in GraphPad Prism (version 9), and differences were considered significant at *p* < 0.05.

## Results and discussion

**Fig. 2:**
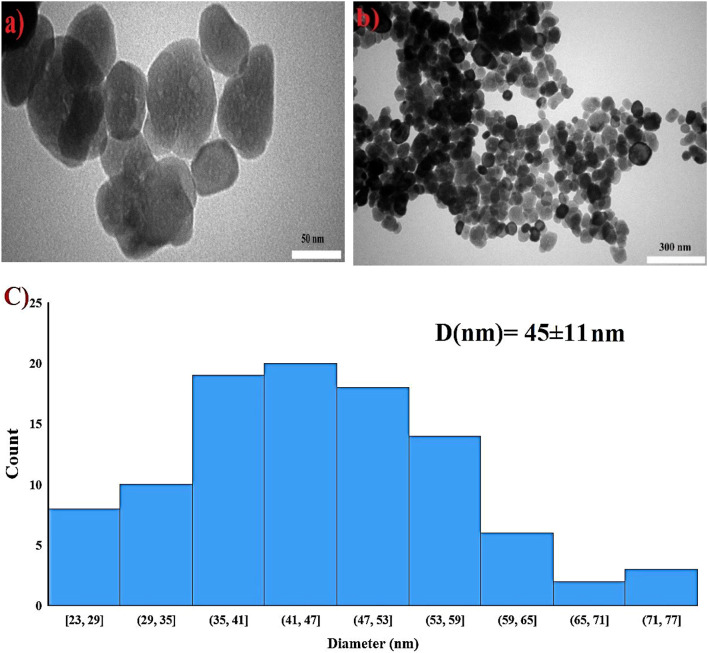
a, b TEM images of α-Fe_2_O_3_ NPs, **c** particle size distribution from TEM images, analyzed using Image J (mean:45 nm; SD: 11 nm).

TEM analysis evaluated the size and morphology of NPs. Figure 2a and b shows images of α-Fe_2_O_3_NPs. In Fig. 2c, the corresponding histogram was plotted using TEM images of the nanoparticles and analyzed with Image J software. The average particle size was estimated to be 45 nm with a standard deviation of 11 nm. Besides, they have a somewhat spherical shape and some porosity on their surface.

**Fig. 3:**
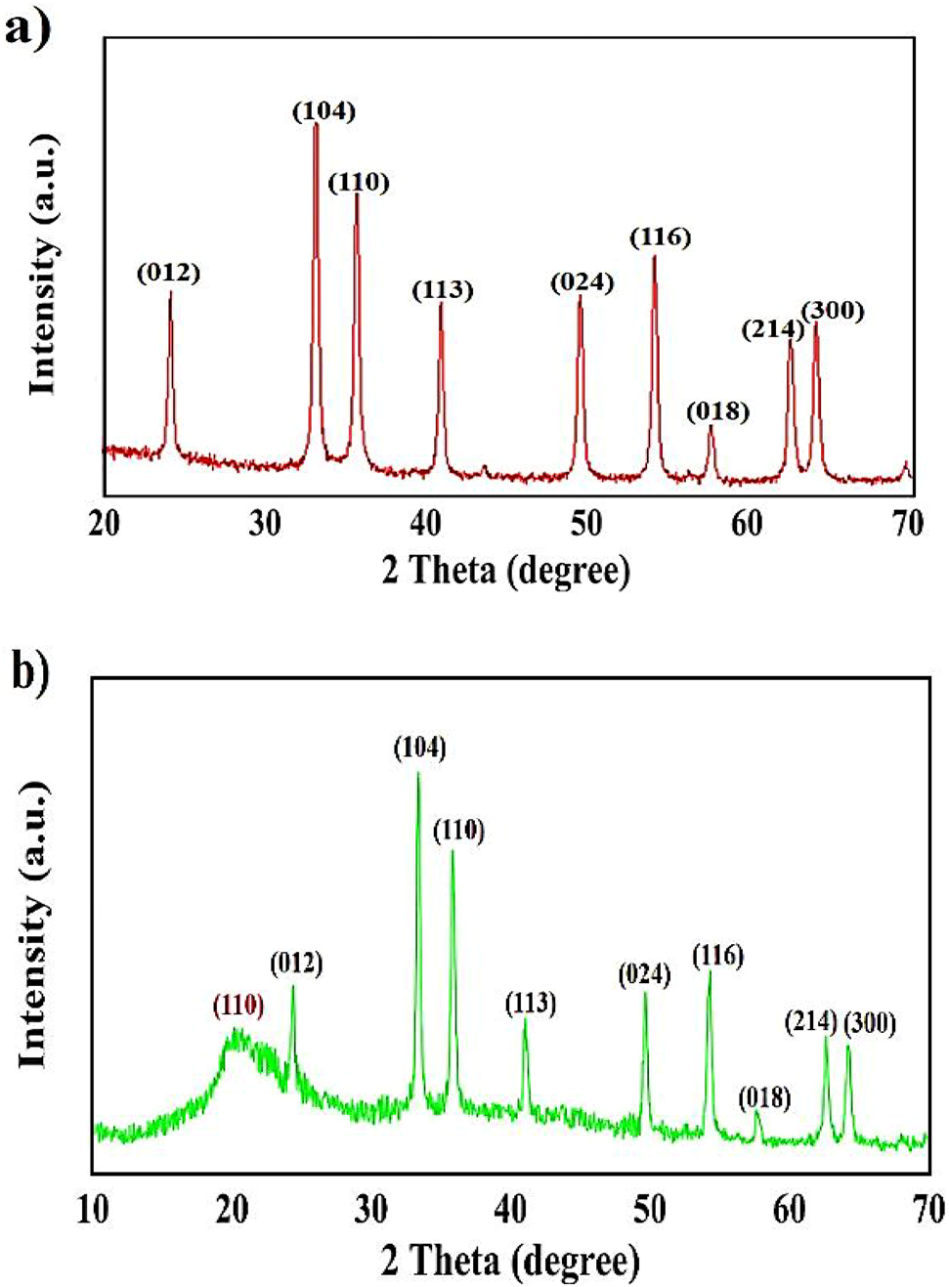
XRD pattern of **a** α-Fe_2_O_3_ NPs, **b** CS-α-Fe_2_O_3_ nanocomposite.

The XRD pattern of α-Fe_2_O_3_NPs is shown in Fig. 3a, which agrees with the standard pattern data (JCPDS card No. 33–0664)^37^. The peaks appearing in the 24.10, 33.09, 35.61, 40.82, 49.44, 54.00, 57.46, 62.41, and 63.95 degrees have been attributed to the diffraction planes (012), (104), (110), (113), (024), (116), (018), (214) and (300) of NPs crystalline respectively. All sharp and narrow peaks appearing in the range of 2θ indicate the crystalline nature and the high purity of α-Fe_2_O_3_NPs extracted by the combined of green synthesis and hydrothermal method. In addition, the XRD pattern of nanocomposite is shown in Fig. 3b. In this pattern, all the characteristic peaks related to α-Fe_2_O_3_NPs and the characteristic broad peak at the angle of 2θ = 20.26 related to CS biopolymer have been observed, confirming the successful formation of this nanocomposite^38^.

The crystallite size of α-Fe_2_O_3_NPs can be estimated by using the Williamson–Hall method (Eq. 1) ^39^:

1 $$\beta \cos \theta =\left( {0.89\lambda } \right)/d+4\varepsilon \sin \theta$$

**Fig. 4:**
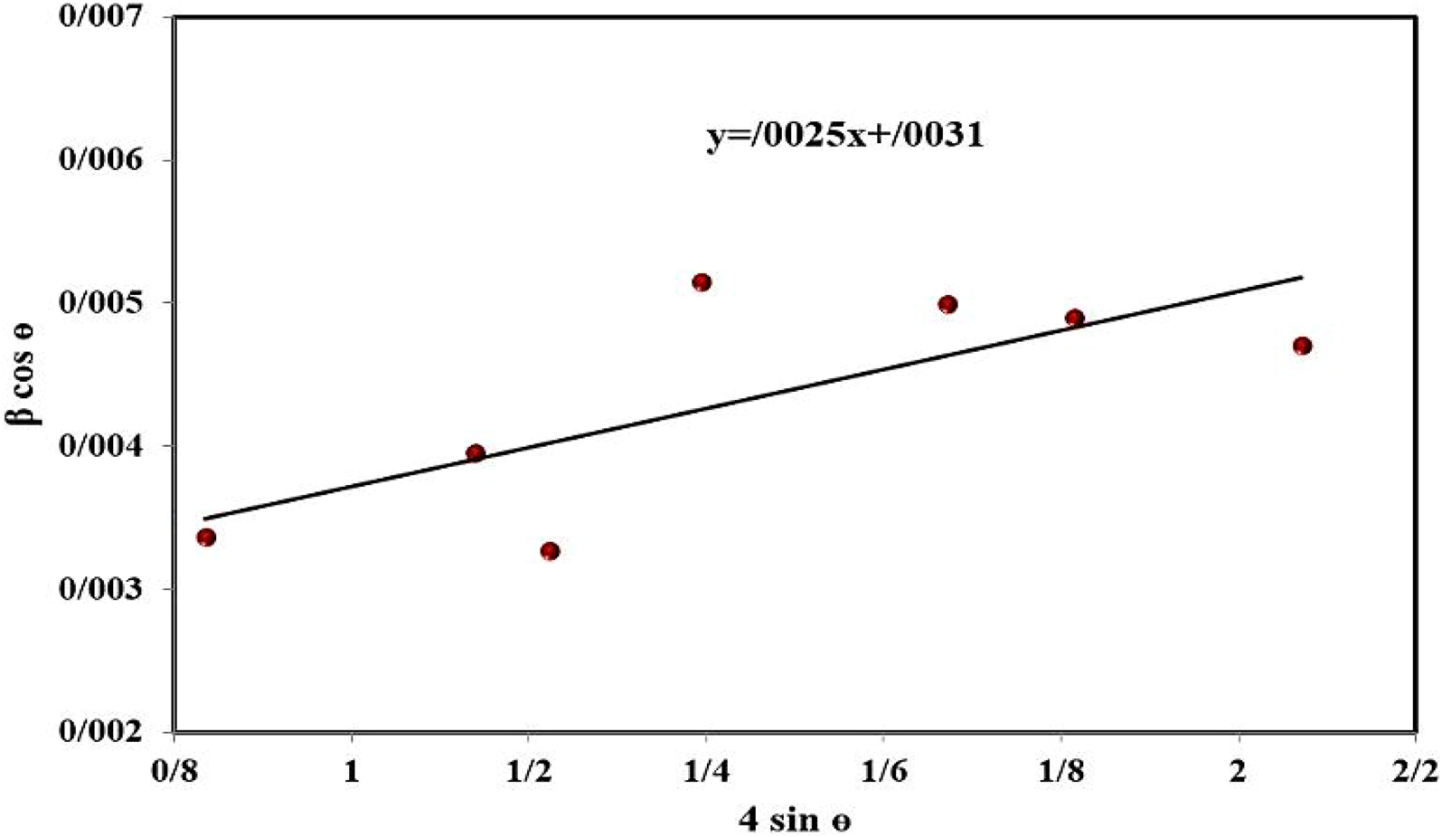
Williamson–Hall diagram of –Fe_2_O_3_ crystallite.

In this equation, β is full width at half maximum (FWHM), θ is the diffraction angle, λ is the X-ray wavelength, d is the average crystallite size, and ε is the lattice strain. The crystallite size of α-Fe_2_O_3_NPs was determined by drawing a diagram (Fig. 4) and calculating the width from the origin. With these interpretations, the crystallite size was estimated to be 43 nm.

**Fig. 5:**
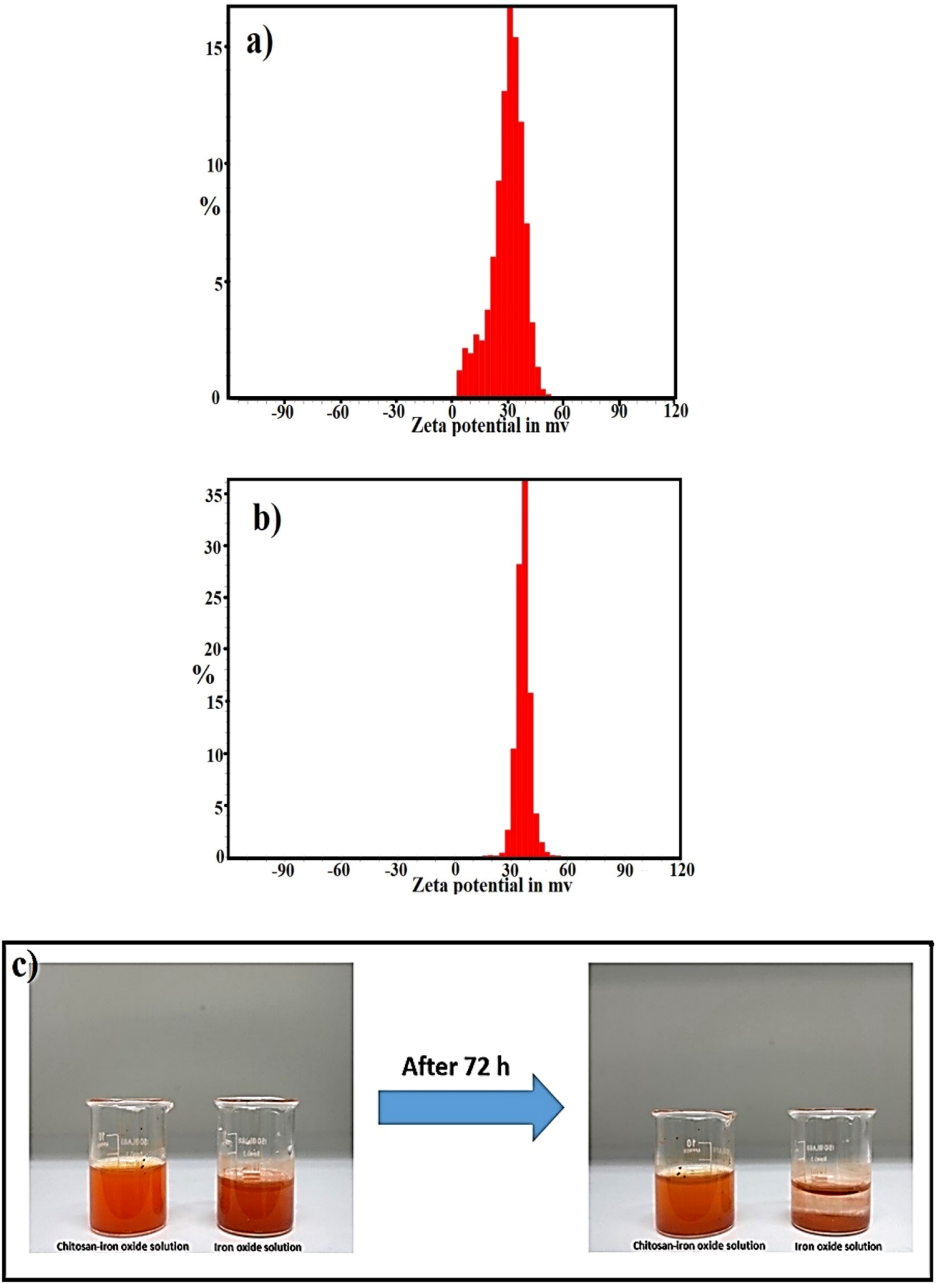
Zeta potential distribution function diagram of **a** α-Fe_2_O_3_ NPs, **b** CS-α-Fe_2_O_3_ nanocomposite, **c** comparing the stability of Fe_2_O_3_ NPs solution and CS-Fe_2_O_3_ nanocomposite solution after 72 h.

One of the important characteristics of colloidal suspensions is the tendency of their particles to stick together. In an aqueous environment, particles continuously interact, and the stability of such solutions is determined based on these interactions. In order to create a stable solution, short-range repulsive forces are needed, among which we can mention the steric interfacial forces that play an important role in stabilizing suspensions. Therefore, these electric forces create layers around the NPs, and this issue plays a significant role in the value of zeta potential and ultimately prevents particles from agglomerating^40^. In this research, the stability of the CS-α-Fe_2_O_3_ nanocomposite solution originates from the strong bonding of CS on the surface and the charge of α-Fe_2_O_3_NPs, which causes repulsion between them. The CS, a biocompatible and biodegradable polymer, forms a protective layer around the α-Fe_2_O_3_NPs, preventing them from agglomerating and thus contributing to the stability of the solution. In order to study the stability of α-Fe_2_O_3_NPs and CS-α-Fe_2_O_3_ nanocomposite solutions with pH 5.11 and viscosity 0.9327 mPas, zeta potential analysis as a critical parameter was performed, and their results are shown separately in Fig. 5a and b. In these graphs, the distribution function of the zeta potential of individual NPs and nanocomposite in the solution is presented as a percentage, and the average zeta potential of unmodified α-Fe_2_O_3_NPs and CS-α-Fe_2_O_3_ nanocomposite was measured 29.93 and 36.67 mV, respectively. Also, by comparing the two graphs, the results indicate that adding CS to α-Fe_2_O_3_NPs narrows the peak width of the graph, which indicates that a large percentage of nanocomposites have the same zeta potential, and this issue was evaluated as stabilization of CS-α-Fe_2_O_3_ nanocomposite suspension. In an experiment, two solutions of NPs and CS-α-Fe_2_O_3_ nanocomposite with the same concentration of 5 mg/ml were prepared, and the role of adding CS in stabilizing the α-Fe_2_O_3_NPs solution after 72 h is shown in Fig. 5c. Another result obtained from the zeta potential of CS-α-Fe_2_O_3_ nanocomposite suspension indicates the net positive charge of these nanocomposites. Furthermore, these nanocomposites can stick and penetrate cancer cells with a negative charge through electrostatic forces^41^.

**Fig. 6:**
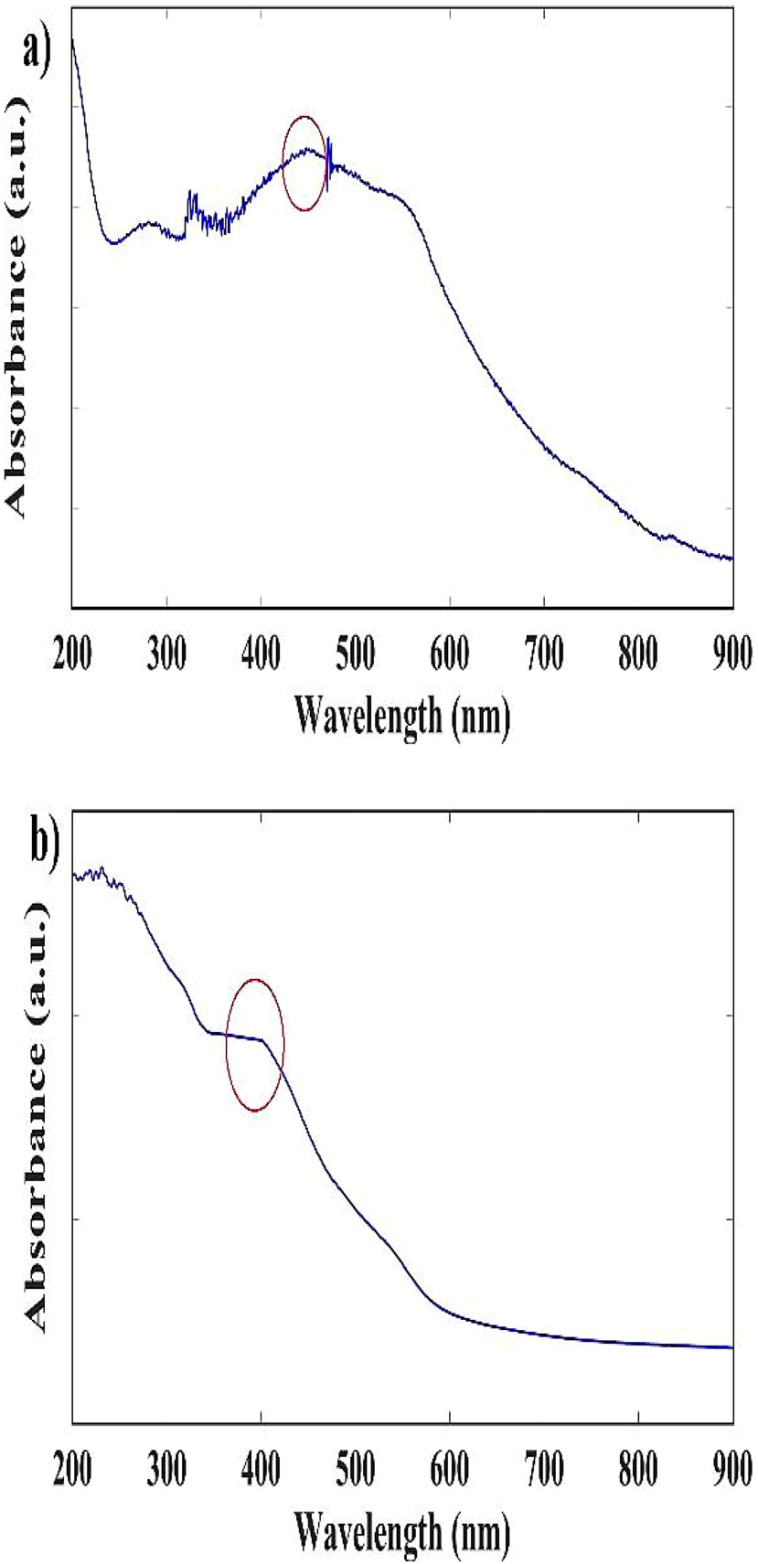
UV–Visible absorption spectrum of **a** α-Fe_2_O_3_ NPs, **b** CS-α-Fe_2_O_3_ nanocomposite.

Figure 6a and b shows the UV–visible absorption spectrum of α-Fe_2_O_3_NPs and CS-α-Fe_2_O_3_ nanocomposite between 200 and 900 nm. The absorption peak for α-Fe_2_O_3_NPs and CS-α-Fe_2_O_3_ nanocomposite is 450 and 380 nm, respectively. This result is significant and reassuring, as it aligns with the data of another research^36^ enhancing our confidence in the findings. Considering the absorption in the infrared region, this issue guarantees the wide potential of this nanocomposite to be used in cancer treatment by PTT and PDT with an 808 nm laser.

The optical band gap of α-Fe_2_O_3_ NPs can be calculated by using Tack’s equation (Eq. 2)^42^.

2 $${(\alpha h\nu )^n}=A\left( {h\nu - {E_g}} \right)$$

**Fig. 7:**
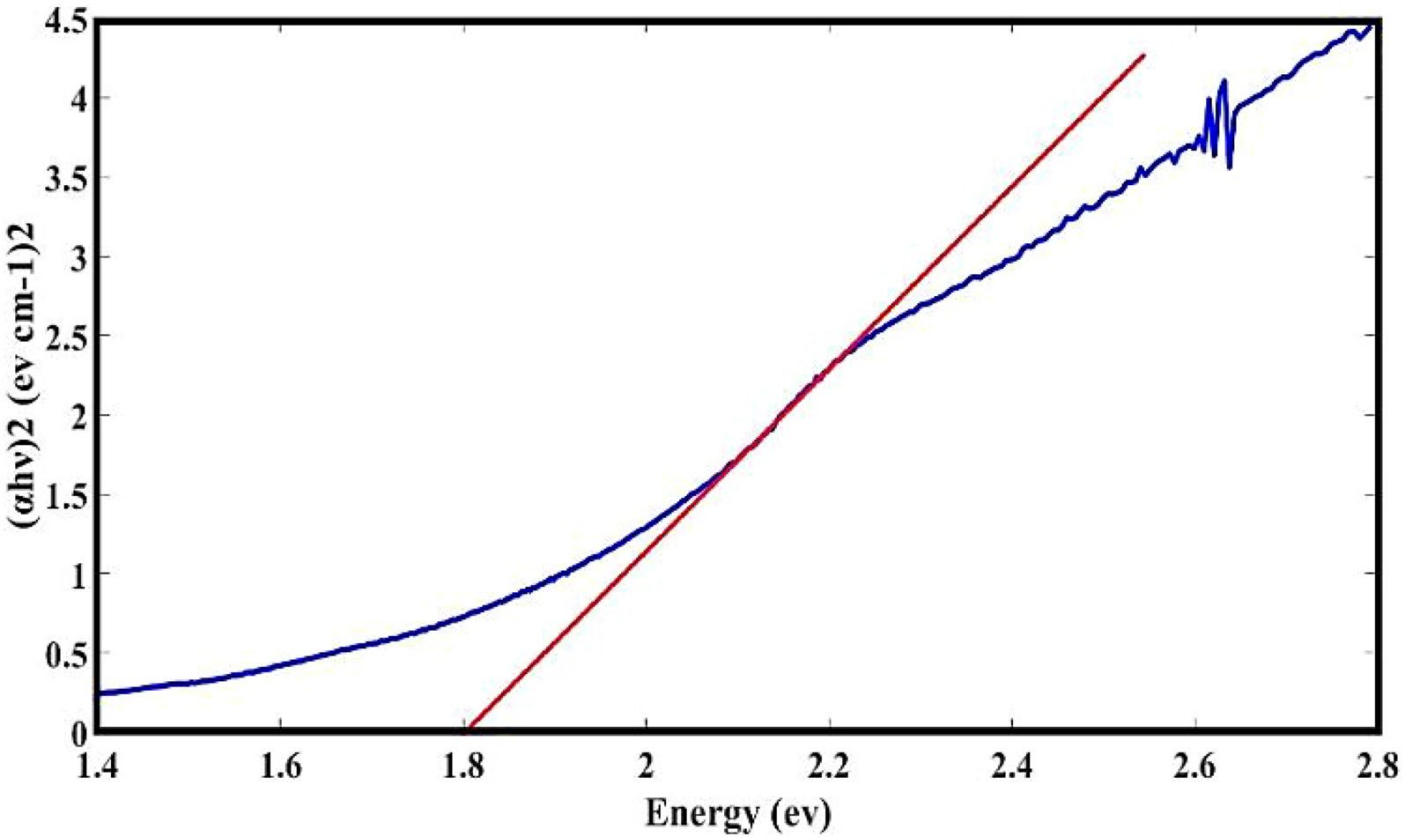
α-Fe_2_O_3_ NPs Tauc diagram.

In this equation, α is the absorption coefficient, A is a constant, hν is the photon energy, and n is a constant that depends on the nature of the electron transition, which is 2 for direct transition and 1/2 for indirect transition. Since α-Fe_2_O_3_ NPs have a direct energy gap, the graph of (αhν)^^2^ in terms of energy is drawn in Fig. 7^43^. The value of the energy gap associated with α-Fe_2_O_3_ NPs was approximated at 1.8 eV using the extrapolation method, which is comparable to the result of another research^44^. Also, our Experimental investigation indicates that the NPs exhibit an indirect band gap of roughly 1.7 eV.

**Fig. 8:**
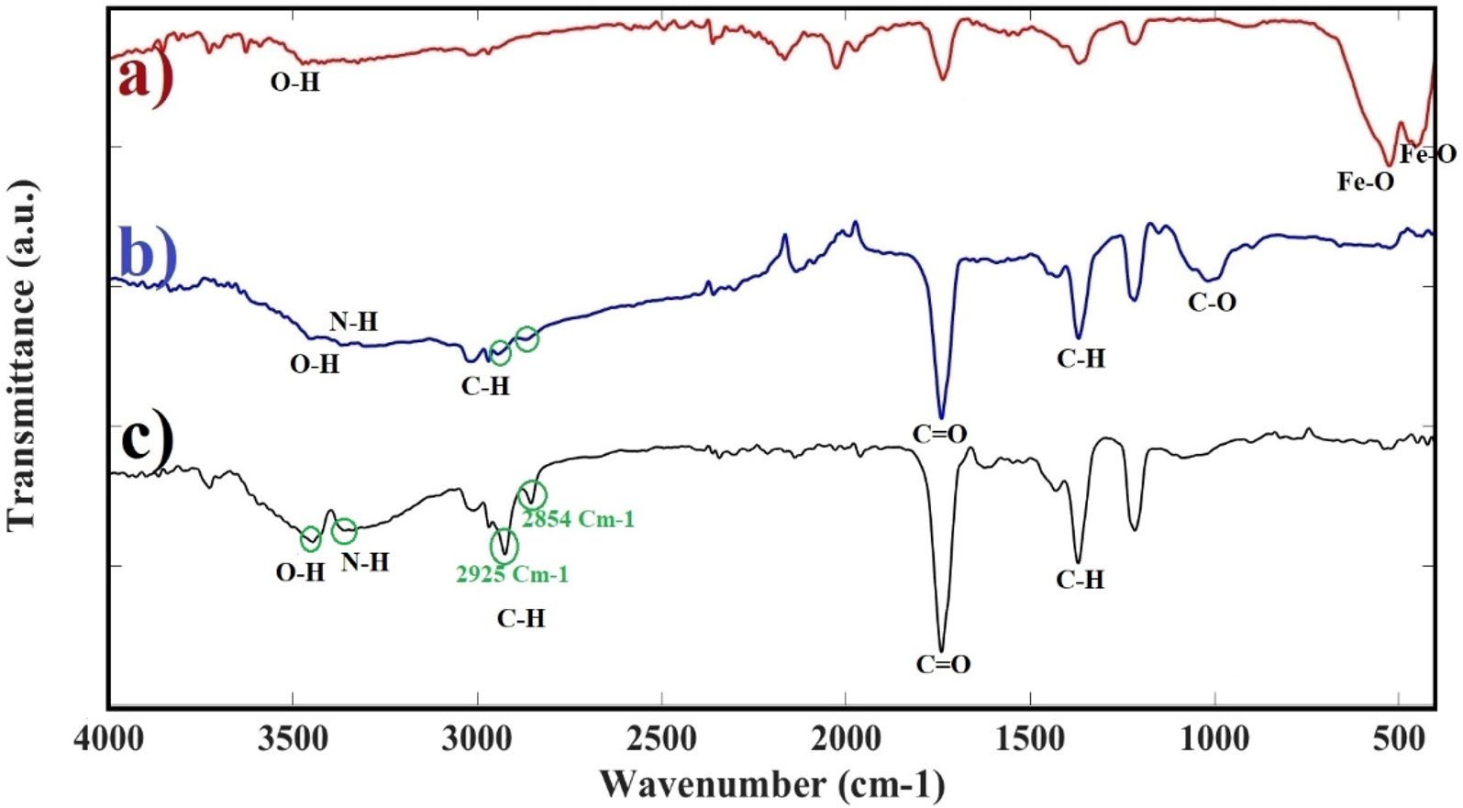
FTIR spectrum of **a** α-Fe_2_O_3_ NPs, **b** CS, **c** CS-α-Fe_2_O_3_ nanocomposite.

FTIR analysis was performed in the 400–4000 cm^− 1^ wave number range to determine the bonds and functional groups on the surface of CS powder, α-Fe_2_O_3_NPs, and CS-α-Fe_2_O_3_ nanocomposite. The FTIR spectrum of α-Fe_2_O_3_NPs is shown in Fig. 8a. A broad peak in the range of 3445 cm^− 1^ is assigned to the stretching vibration between oxygen and hydrogen belonging to the functional groups of hydroxyl and water molecules, which proves the absorption of some water at the surface of α-Fe_2_O_3_NPs^45^. Also, two sharp absorption peaks in the range below 1000 Cm^− 1^ indicate the main characteristics of α-Fe_2_O_3_ NPs, which are attributed to the stretching frequencies of metallic iron. The high frequency peak in 526 cm^− 1^ refers to Fe-O deformation in tetrahedral and octahedral environments. At the same time, the peak at low frequency in the range of 450 cm^− 1^ refers to the Fe–O shape change in the octahedral environment of hematite^46^. In the spectrum of CS powder, the peaks appearing in the 3445 cm^− 1^ and 3360 cm^− 1^ range are related to O–H and N–H stretching vibrations, respectively, and intra molecular hydrogen bonds. The peaks around 2925 cm^− 1^ and 2854 cm^− 1^ are attributed to symmetric and asymmetric C–H stretching vibrations, which are common bonds characteristic of polysaccharides. The remaining presence of *N*-acetyl groups was confirmed by peaks in the range of 1741 cm^− 1^ related to C=O stretching vibrations. Also, C–H bending vibrations and the symmetric deformation were confirmed by the peak in 1369 cm^− 1^. The peak appearing in 1020 cm^− 1^ is attributed to C–O stretching vibrations. All peaks related to CS material are shown in Fig. 8b^47^. By analyzing the FTIR analysis of CS-α-Fe_2_O_3_ nanocomposite, all the characteristic peaks of the infrared Fourier transform of α-Fe_2_O_3_NPs except Fe–O and all the characteristic peaks of CS were observed (Fig. 8c).

**Fig. 9:**
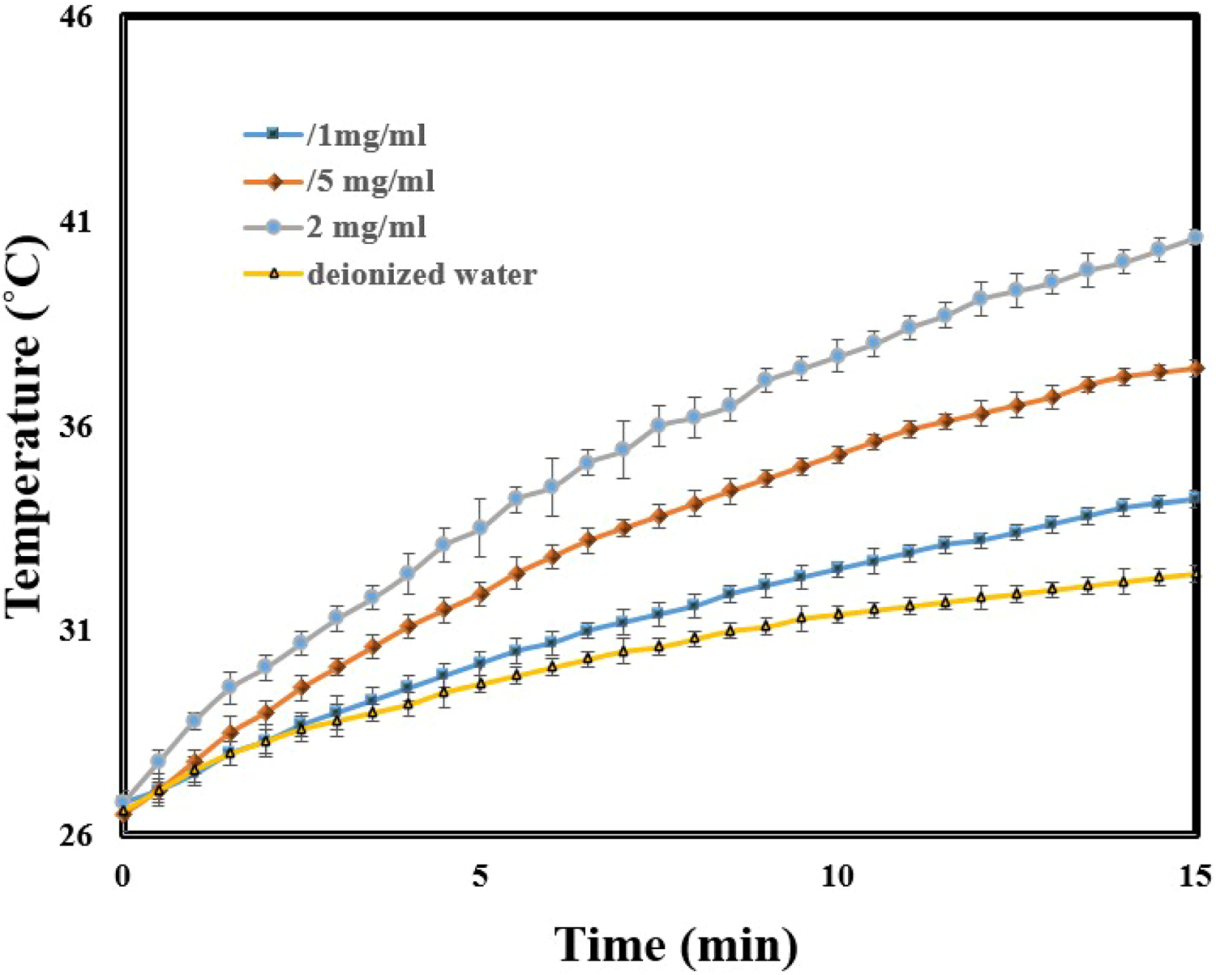
Temperature change diagram according to 808 nm laser irradiation time CS-α-Fe_2_O_3_ nanocomposite solution in different concentrations. Data are presented as mean ± SD (*n* = 3). Error bars represent standard deviation.

Three different concentrations of CS-α-Fe_2_O_3_ nanocomposite solution were prepared to study PTT effects using an ultrasonic bath. Three ml of each of these different concentrations were poured into a tube and placed in a relatively dark under the radiation of 808 nm laser with a power density of 1 W/cm^2^. The temperature change was measured by a digital thermometer equipped with a thermal sensor for 15 min. Figure 9 shows temperature changes over 15 min for nanocomposite solutions at 0.1, 0.5, and 2 mg/ml concentrations, and DI water, with increases of 7.4, 10.9, 13.8, and 5.8 °C, respectively. Data are presented as mean ± SD (*n* = 3), with error bars indicating standard deviation. Detailed temperature differences for each concentration are provided in Table 1 .Due to the appropriate temperature change and low concentration, the 0.5 mg/ml concentration was determined as the optimal concentration.

**Table 1:** PTT details for CS-α-Fe_2_O_3_ nanocomposite and DI water under 808 nm laser irradiation.

Temperature change (°C )	Final temperature (°C )	Initial temperature (°C )	Concentration (mg/ml)	material
7.4	34.2	26.8	0.1	
10.9	37.4	26.5	0.5	Nano composite solution
13.8	40.6	26.8	2	
5.8	32.4	26.6	-	DI water

The photothermal conversion efficiency of the CS-α-Fe_2_O_3_ nanocomposite was calculated using Roper’s method under 808 nm laser irradiation. Roper’s equation models the photothermal process as a balance between the heat generated by light absorption and the heat lost to the surroundings, allowing photothermal conversion efficiency to be derived from the steady-state temperature and system parameters^48^. In the experiment, the temperature of the nanocomposite solution increased from 24 °C to a saturated value of 42.1 °C, while DI water under identical conditions reached only 34.2 °C. Based on this temperature rise and heat transfer analysis, the efficiency was determined to be 7%. Detailed calculations, fitting procedures, and related graphs are provided in the Supplementary Information.

**Fig. 10:**
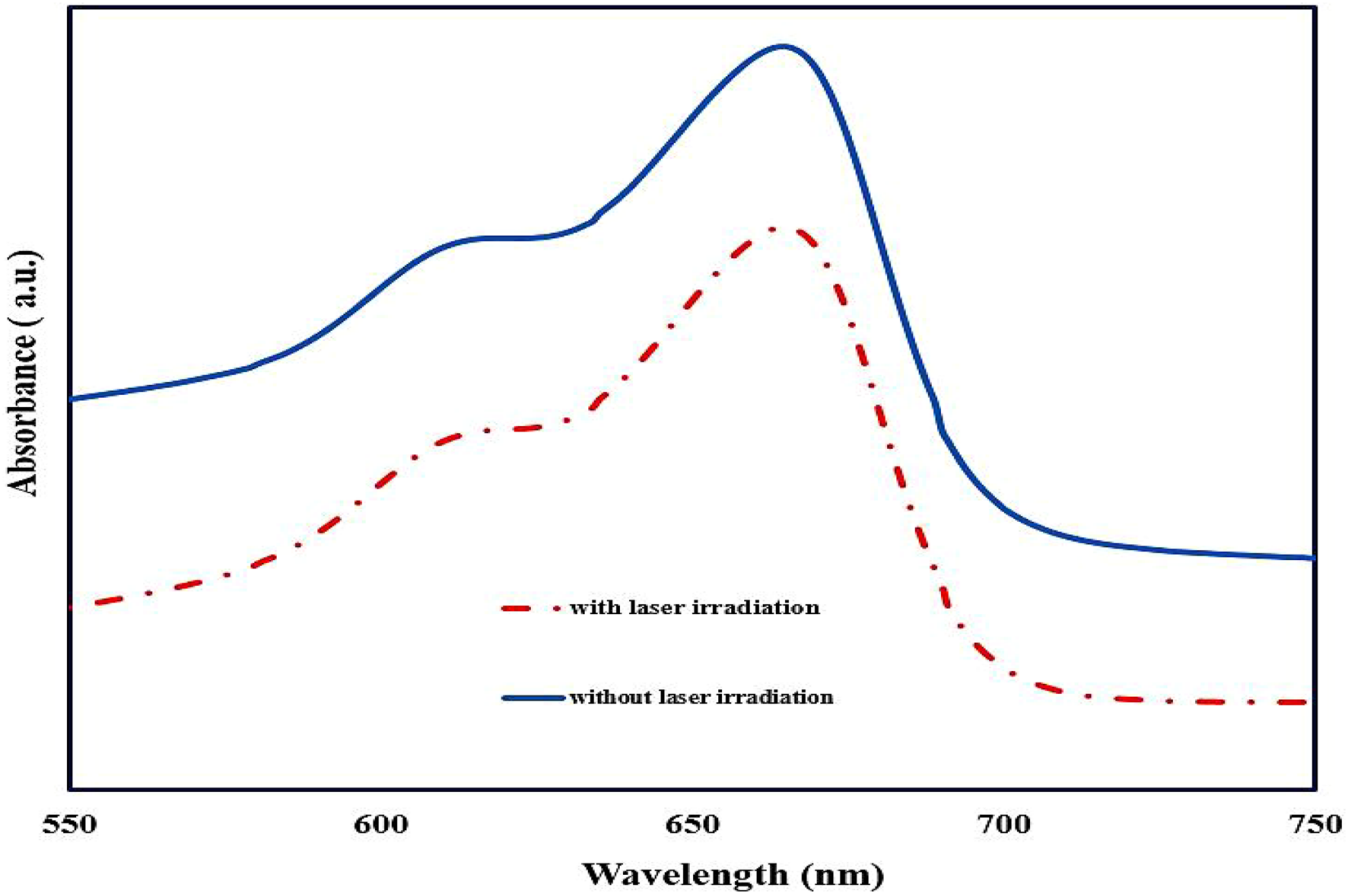
Methylene blue absorption spectrum before and after 808 nm laser irradiation for α-Fe_2_O_3_NPs.

When α-Fe_2_O_3_NPs as semiconductors are exposed to laser irradiation, the photocatalytic process is activated, in which the photon energy is used to transfer electrons from the valence band to the conduction band, and at the same time, it creates a similar number of holes in the valence band, which ultimately leads to the formation of an electron-hole pair. Continuing this separation and transfer of charges to the surface leads to oxidation and reduction reactions with the molecules around these α-Fe_2_O_3_NPs, which can produce ROS. By studying previous research, the ability of α-Fe_2_O_3_NPs to create hydroxyl ROS was determined^35^. Therefore, methylene blue was used as a hydroxyl radical probe to detect indirectly this type of free radical. For this purpose, in an experiment, 1 mg of NPs powder was dissolved in 1.5 ml of DI water and 0.5 ml of methylene blue (0.2 mg/ml) using an ultrasonic bath. Two similar samples were prepared, and in a relatively dark room, 3 ml of each sample was placed on magnetic stirrers, and only one sample was exposed to an 808 laser with a power density of 1 W/cm^2^ for 10 min. Then, the methylene blue absorption spectrums of two samples were measured by a spectrometer, and the reduction of the characteristic absorption peak of methylene blue in the sample under laser irradiation compared to the non-irradiated sample at the wavelength of 664 nm was observed. The results are shown in Fig. 10. The electrons and holes created by the photocatalytic process provide the conditions for producing active oxygen species around the α-Fe_2_O_3_NPs. In this way, the holes formed in α-Fe_2_O_3_NPs lead to the oxidation of the methylene blue molecule, which turns it into an active substance that is ready to react. Also, the electrons on the surface of α-Fe_2_O_3_NPs are transferred to oxygen molecules dissolved in water, which produces superoxide radical negative ions. These ions, after reacting with water molecules, can decompose, and consequently, hydroperoxyl radicals and hydroxyl ions are produced. In the end, after reacting hydroperoxyl radicals with water molecules and proton absorption, this process produces hydroxyl radicals as Eq. (3)^49^:

3 $$\begin{array}{*{20}{c}} {\alpha - F{e_2}{O_3}+h\nu \to \alpha - F{e_2}{O_3}+{e^ - }+{h^+}} \\ {{h^+}+MB \to M{B^{ \cdot +}}} \\ {{O_2}+{e^ - } \to O_{2}^{{ \cdot - }}} \\ {O_{2}^{{ \cdot - }}+{H_2}O \to HO_{2}^{ \cdot }+O{H^ - }} \\ {HO_{2}^{ \cdot }+{H_2}O \to O{H^ \cdot }+{H_2}{O_2}} \end{array}$$

To assess the cytotoxicity of the CS-α-Fe_2_O_3_ nanocomposite, an MTT assay was carried out on AGS gastric cancer cells under in vitro conditions. This experiment includes three independent biological replicates, each containing five technical replicates. The percentage of relative cell viability was determined by calculating the ratio of treated cell viability to that of untreated control cells. Results are expressed as the mean of three biological replicates (*n* = 3) ± standard deviation. Statistical analysis was carried out using GraphPad Prism (version 9), with group comparisons performed via analysis of variance (ANOVA) followed by Tukey’s post hoc test. A p value < 0.05 was deemed statistically significant. These Cells were treated with two concentrations of the nanocomposite: 250 ppm (Group 1) and 500 ppm (Group 2). Each concentration was tested under two conditions—with and without irradiation using an 808 nm laser (power density: 1 W/cm², duration: 15 min). Untreated cells were used as the negative control, while a separate control group consisting of cells exposed to laser alone (without the nanocomposite) was included to isolate the impact of irradiation itself.

**Fig. 11:**
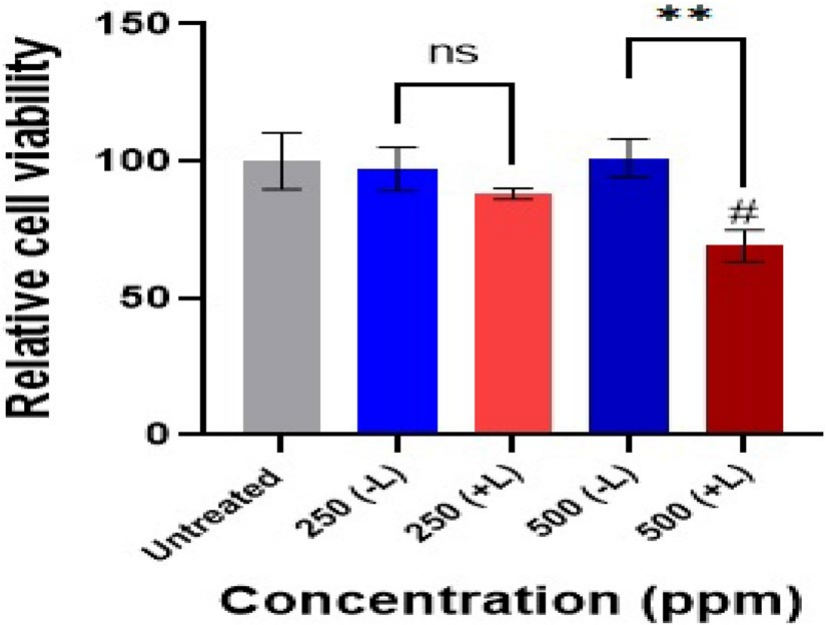
Effect of CS-nanocomposite at two concentrations (250 ppm and 500 ppm), with and without 808 nm laser irradiation, on the viability of AGS cells as determined by the MTT assay. The data are presented as the mean of three independent experiments ± standard deviation. Comparisons among multiple groups were performed using one-way ANOVA followed by Tukey’s post hoc test (ns = non-significant, ** = *p* < 0.01, # = statistically significant compared to untreated cells).

In Group 1, treatment with 250 ppm of the nanocomposite in the absence of laser exposure resulted in minimal cytotoxicity, with a high cell viability of 97%. However, upon laser irradiation, viability decreased slightly to 88%. In Group 2, treatment with 500 ppm of the nanocomposite without irradiation maintained a similarly high viability of 95%, indicating low inherent toxicity. In contrast, laser-irradiated cells in this group showed a significant decrease in viability to 68%, suggesting a pronounced phototoxic effect. The corresponding results are presented in Fig. 11.

The 250 and 500 µg/mL non-laser groups did not show any statistically significant cytotoxicity compared to the control group. Moreover, at the concentration of 250 µg/mL, there was no statistically significant difference between the laser and non-laser treated groups (ns). However, at 500 µg/mL, a statistically significant difference was observed between the laser and non-laser groups. These findings highlight the biocompatibility of the nanocomposite in the absence of laser activation, even at higher concentrations. Conversely, under laser exposure, a concentration-dependent reduction in cell viability was observed, demonstrating the nanocomposite’s potential for application in PTT and PDT therapy.

## Conclusion

In this research, biocompatible α-Fe_2_O_3_NPs were synthesized as a photo-agent for cancer treatment by combining green and hydrothermal synthesis. The influential role of CS coating as a biological polymer around NPs in creating the stability of the suspension was determined by zeta potential analysis. The optical and structural properties of NPs were investigated in detail by XRD, TEM, FTIR, and UV-visible. The band gap was approximated at 1.8 eV. The absorption spectra of NPs show a high potential for applying PTT and PDT methods in the infrared range. The effects and efficiency of PT conversion of Cs-α-Fe_2_O_3_ nanocomposite were evaluated under 808 laser radiation, and the concentration of 5 mg/ml was determined as the optimal concentration to destroy cancer cells. The PT conversion efficiency of 7% for nanocomposite was calculated using the PT. The ability of α-Fe_2_O_3_NPs to create ROS was determined using a methylene blue probe. The decrease in the characteristic absorption of methylene blue via α-Fe_2_O_3_NPs under an 808 nm laser irradiation with a power density of 1 W/cm^2^ for 10 min was considered proportional to the hydroxyl produced. The results obtained from this research demonstrated the remarkable potential of the CS-α-Fe_2_O_3_ nanocomposite as a photoactive agent for the combined implementation of PTT and PDT, further validated by MTT assay results indicating about a 12% reduction in cell viability. While these findings are encouraging and point toward exciting possibilities in precision cancer treatment, further comprehensive studies are required to confirm its efficacy and ensure its safe translation into clinical practice.

## Supplementary Information

Below is the link to the electronic supplementary material.

Supplementary Material 1

## Data Availability

The datasets used and/or analyzed during the current study are available from the corresponding author on reasonable request. Correspondence should be addressed to Hossein Shirkani at shirkani@pgu.ac.ir **.**.
