# Supplementary material for: Facile and green synthesis of α-Fe2O3 nanoparticles stabilized with chitosan for phototherapy with 808 nm laser irradiation
Source: Sci Rep. 2025 Sep 2;15:32269. doi: 10.1038/s41598-025-17797-2 (PMC12402456; doi:10.1038/s41598-025-17797-2)
Supplement: Supplementary file 1 — Supplementary Material 1 [file 41598_2025_17797_MOESM1_ESM.docx]

**Supplementary Information**

**Facile and green synthesis of** $\boldsymbol{\alpha-}\mathbf{Fe}_{\boldsymbol{2}}\mathbf{O}_{\boldsymbol{3}}$**nanoparticles stabilized with chitosan for phototherapy with 808 nm laser irradiation**

Farshad Dehdashti^1^, Hossein shirkani^1^,*, Mohsen Mehrabi^1^, Amirhossein Ahmadi^2^

^1^ Physics Department, Persian Gulf University, Bushehr, Iran, P. O. Box: 7516913817

^2^Department of Biological Science & Technology, Persian Gulf University, Bushehr, Iran, P. O. Box: 7516913817

* Email: [shirkani@pgu.ac.ir](mailto:shirkani@pgu.ac.ir)

**Calculation of the photothermal conversion efficiency**

In this study, Roper's equation (equation 1) was used to calculate the photothermal conversion efficiency of nanocomposite.

| $\sum m_{i}C_{pj}dT/dt=Q_{NC}+Q_{\text{dis }}-Q_{\text{surr }}$ | (1 |
| --- | --- |

In this research, the tube containing CS-$\alpha-\mathrm{Fe}_{2}O_{3}$ nanocomposite solution was considered a thermodynamic system and equation 1 was considered as a balanced relationship between the input and output energies of this system. In this equation, m and C are the mass and heat capacity of the components of the solution respectively, T is the temperature of the solution, $Q_{NC}$ is the amount of heat energy given to the system per unit of time by the nanocomposite and $Q_{\text{dis }}$is the amount of energy lost heat per unit of time by the wall of the tube and water molecules after absorbing light and $Q_{\text{surr }}$is the amount of energy transferred per unit of time between the studied system and its surroundings. In this research, the amount of mass nanocomposite in the solution was omitted due to its small amount compared to the mass of water. Equation 2 was used to calculate the value of$Q_{NC}$, which is the heat lost by electron-phonon relaxation Plasmon on the surface of the nanocomposite after absorbing an 808 nm laser.

| (2 | $Q_{NC}=I\left( 1-{10}^{-A808} \right)\eta$ |
| --- | --- |

I is the laser power, η is the PT conversion efficiency, and A808 is the absorption of nanocomposite at the wavelength of 808 nm. Also, equation 3 was used to calculate $Q_{\text{Surr }}$ of the heat exchanged between the solution and its surroundings.

| (3 | $Q_{\text{Surr }}=hS\left( T-T_{\text{surr }} \right)$ |
| --- | --- |

In equation 3, a linear relationship between the amount of heat energy exchanged and the temperature difference between the solution and the environment is shown, where h is the heat transfer coefficient, S is the container's surface area, and $T_{\text{surr }}$is the surrounding environment's temperature. We can conclude that with the increase in the temperature difference between the solution and its surrounding environment, the exchanged heat energy also increases. The amount of energy input to system ${(Q}_{\text{dis }}$+$Q_{NC})$ was limited because the laser power was defined. Therefore, with time, the temperature of the solution under the laser radiation reached a constant value, and the left side of equation 1 became zero. Finally, the system reached a temperature saturation state where the sum of the two energy inputs was equal to the $Q_{\text{Surr }}$value. Finally, equation 4 was obtained to calculate the PT conversion efficiency of the nanocomposite.

| (4 | $\eta=\frac{hS\left( T_{\text{max }}-T_{\text{surr }} \right)-Q_{Dis}}{I\left( 1-{10}^{-A808} \right)}$ |
| --- | --- |

3 ml of nanocomposite solution was poured into a tube, placed in a dark room with a temperature of 24°C and irradiated under an 808 nm laser with a power density of 1 W/cm^2^. The temperature of the solution reached the maximum saturated value of 42.1 °C (Fig. S1a). In order to calculate $hS$ in equation 4, the dimensionless parameter $\theta$ based on the temperature saturation temperature (equation 5) and $\tau_{s}$ as the time constant of the system (equation 6) were defined:

| (5 | $\theta=\frac{\left( T-T_{\text{surr }} \right)}{\left( T_{max}-T_{\text{surr }} \right)}$ |
| --- | --- |

| (6 | $\tau_{s}=\frac{\sum_{i} m_{i}C_{pj}}{hS}$ |
| --- | --- |

By inserting equations 5 and 6 in equation 1 and arranging the sentences, equation 7was obtained.

| (7 | $\frac{d\theta}{dt}=\frac{1}{\tau_{s}}\left( \frac{Q_{NC}+Q_{Dis}}{hS\left( T_{\text{max }}-T_{\text{surt }} \right)}-\theta\right)$ |
| --- | --- |

After reaching the temperature saturation of the system, the laser was turned off and the value of ${(Q}_{\text{dis }}$+$Q_{NC})$ becomes zero, and after a minor mathematical operation, equation 8 was obtained:

| *(8)* | $t=-\tau_{s}ln \theta$ |
| --- | --- |

*
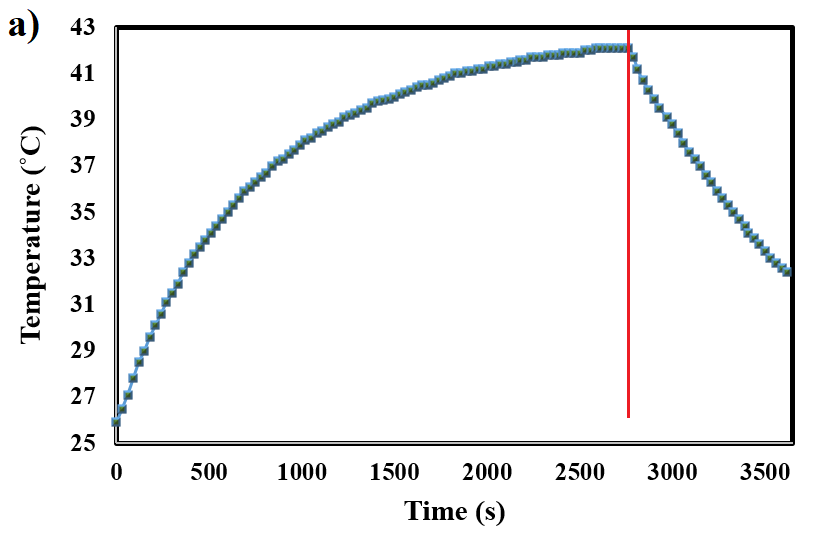
*

*
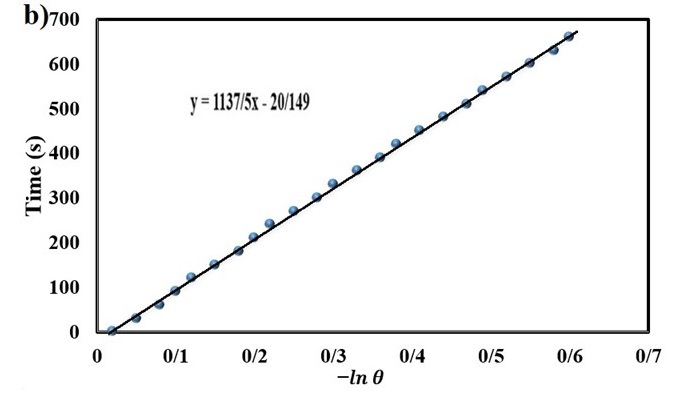
*

**Fig. S1. (a) Temperature change in terms of time CS-**$\boldsymbol{\alpha-}\mathbf{Fe}_{\boldsymbol{2}}\mathbf{O}_{\boldsymbol{3}}$ **nanocomposite solution diagram before and after turning off the 808 nm laser (b) t in terms of** $\boldsymbol{-ln \theta}$ **diagram to calculate** $\boldsymbol{\tau}_{\boldsymbol{s}}$ **related to CS-**$\boldsymbol{\alpha-}\mathbf{Fe}_{\boldsymbol{2}}\mathbf{O}_{\boldsymbol{3}}$ **nanocomposite.**

The value of $\tau_{s}$was calculated 1137.5 seconds by measuring the slope of diagram t in terms of -ln θ, (Fig. S1b). By using equation 6 and considering the heat capacity of water (4.2 J/g) and the mass of water in the solution (3 g), $\mathrm{hS}$ value was calculated 0.011 J/s °C. To calculate the value of$Q_{Dis}$, 3 ml of deionized (DI) water was poured into a test tube without considering the nanocomposite and placed in a relatively dark room at a temperature of 24 °C exposed to an 808 nm laser with a power density of 1 W/cm^2^. After 1950 seconds, the temperature of the solution reached the maximum saturated value of 34.2 °C (Fig. S2a). In such a case, by removing the value of $Q_{NC}$and in the state of temperature saturation, equation 4 was converted into equation 9. Here, as in the previous step, in order to calculate $\mathrm{hS}$ using the newly defined parameters θ and $\tau_{s}$, after turning off the laser and in the cooling phase of the distilled water, the value of $\tau_{s}$was calculated 898 seconds by measuring the slope of diagram t in terms of -ln θ (Fig.S2b). Using equation 6, the value of $hS$ was 0.014 J/s °C, and by inserting, the obtained numbers in equation 9, the value of $Q_{Dis}$was calculated as 0.143 J/s.

| (9 | $Q_{Dis}=Q_{\text{Surr }}=hS\left( T_{\text{max }}-T_{\text{surr }} \right)$ |
| --- | --- |

*
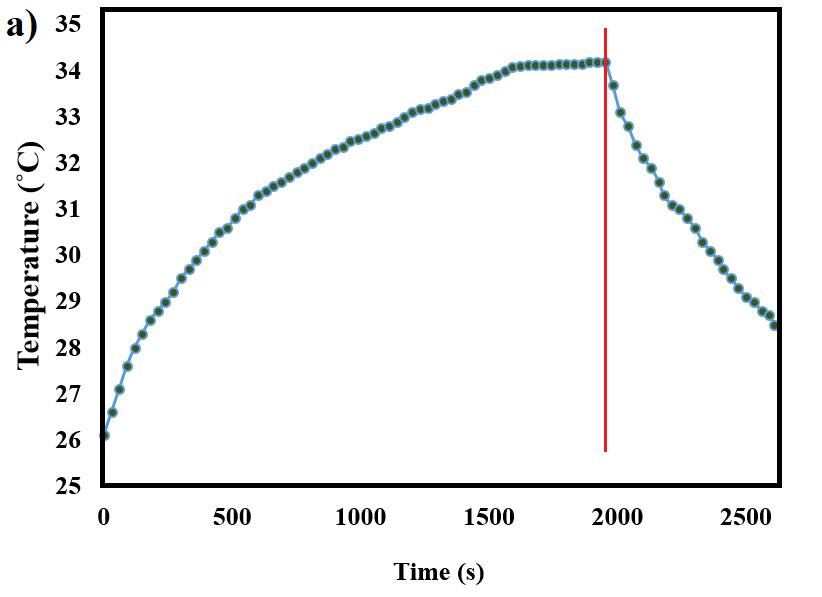
*

*
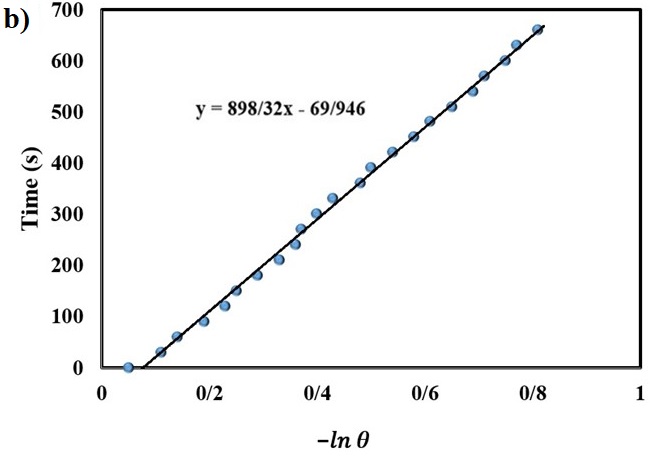
*

**Fig. S2. (a) Temperature change in terms of time related to DI water diagram before and after turning off the 808 nm laser (b) Tin terms of** $\boldsymbol{-ln \theta}$ **diagram to calculate** $\boldsymbol{\tau}_{\boldsymbol{s}}$ **related to DI water**

The value of PT conversion efficiency of CS-$\alpha-\mathrm{Fe}_{2}O_{3}$ nanocomposite was calculated 7% by inserting all calculated parameters on the right side of equation 4.
